# Influencing mechanisms of undergraduates’ continuous learning behavior in the context of intelligent technology: an empirical analysis

**DOI:** 10.3389/fpsyg.2026.1906549

**Published:** 2026-10-01

**Authors:** Dingpu Shi, Ying Wei, Duo Yang

**Affiliations:** 1 Faculty of Education, Yunnan Normal University, Kunming, China; 2 The Faculty of Education, Southwest University, Chongqing, China

**Keywords:** continuous learning behavior, hidden markov model, intelligent learning environments, support vector regression, undergraduates

## Abstract

**Introduction:**

This study investigates undergraduates’ continuous learning behavior in intelligent-technology-supported university classrooms. Continuous learning behavior is conceptualized as observable persistence across repeated learning occasions. Drawing on Expectation-Confirmation Theory (ECT) and the Stimulus-Organism-Response (S-O-R) framework, the study examines how technology-enabled instructional and social features are associated with learners’ affective and cognitive processes and their subsequent learning-state transitions.

**Methods:**

Data were obtained from 2,133 undergraduates at M University across the 2021–2023 College and University Student Survey (CCSS) waves, comprising 70,280 recorded observations, with an average of 32.95 observations per participant. Survey-derived and system-captured indicators were analyzed using Hidden Markov Models (HMMs) to identify latent continuous learning states and transition patterns. Models containing one to eight states were compared, and Support Vector Regression (SVR) was used exploratorily to examine the associations of affective identification, immersion, satisfaction, and learning attention with the identified learning states.

**Results:**

The three-state HMM yielded the lowest Bayesian Information Criterion (BIC = 3,326.39) and was therefore selected as the optimal specification. The three latent states were interpreted as emerging persistence, established persistence, and high persistence. Exploratory SVR results showed that learning attention had the strongest positive normalized association (0.751), followed by satisfaction (0.434). Affective identification showed a negative overall association (-0.123), although a small positive coefficient was observed in State 2 (0.027), providing only limited state-dependent support for H1. Immersion was negatively associated overall (-0.187) and showed a particularly strong negative coefficient in State 3 (-0.473); thus, H2 was not supported as a uniform positive relationship.

**Discussion:**

The findings suggest that continuous learning behavior is better understood as a dynamic, state-dependent process rather than as a fixed learner characteristic or developmental stage. Learning attention and satisfaction appear to be more consistently associated with persistent learning states than affective identification or immersion. Given the observational design and single-institution sample, the findings should be interpreted as associational rather than causal.

## 1 Introduction

Intelligent learning environments increasingly integrate learning management systems (LMSs), learning analytics, data-informed content, and technology-mediated interaction to make learning activities more visible and responsive. Reviews indicate that these environments can extend access to timely feedback and personalized support, but their educational value depends on pedagogical alignment, usability, infrastructure, and learners’ capacity to act on system information (Tabuenca et al., 2021; Dimitriadou and Lanitis, 2023; Mhlongo et al., 2023). Technology availability alone therefore cannot explain sustained participation. A more precise account must examine how learners appraise concrete instructional and social features and how those appraisals are associated with observable behavior across repeated learning occasions.

The Stimulus-Organism-Response (S-O-R) framework provides this process logic: environmental stimuli shape approach or avoidance responses through individuals’ internal cognitive and affective states (Mehrabian and Russell, 1974). In the present LMS-centered setting, the stimuli are LMS content, analytics feedback, and teacher/peer interaction; the organism component comprises affective identification, immersion, satisfaction, and attention; and the response comprises continuous learning behavior and latent behavioral states. Expectation-Confirmation Theory (ECT) complements this structure by explaining post-adoption appraisal: confirmation of prior expectations influences perceived usefulness and satisfaction, which are central to continued system use (Bhattacherjee, 2001). Research on virtual classrooms and higher-education e-learning likewise identifies usefulness, satisfaction, and confirmation-related evaluations as important correlates of continuance (Alshammari and Alshammari, 2024; Ullah et al., 2023). Integrating ECT with S-O-R thus links specific technology-enabled conditions to internal appraisals and repeated behavioral responses. Because the present design is observational, these relationships are treated as associations rather than causal pathways.

A central conceptual issue is that continuance intention, learning engagement, and continuous learning behavior are sometimes discussed as though they were interchangeable. Continuance research generally examines learners’ willingness or intention to keep using an online platform (Zhu et al., 2020; Wang et al., 2023; Dai et al., 2024), whereas engagement concerns the cognitive, affective, and behavioral intensity of participation within a learning experience (Kimbark et al., 2017; Ma et al., 2024). Neither construct is identical to actual persistence across a sequence of observed occasions. Accordingly, this study defines continuous learning behavior as repeated, observable participation and persistence across ordered learning occasions. Affective identification consistently denotes learners’ felt alignment with and acceptance of the technology-mediated teaching process. These definitions prevent the focal behavioral outcome and affective mechanism from being conflated with adjacent constructs.

A second gap concerns temporal heterogeneity. Cross-sectional models can estimate average relationships but cannot show whether learners occupy qualitatively different persistence states or switch between them. Process-oriented studies indicate that regulation and participation may change across successive online-learning episodes (Zhang et al., 2021), while trajectory research emphasizes the value of repeated observations for distinguishing stable differences from within-person change (Herle et al., 2020). Hidden Markov models can infer unobserved states from ordered indicators and characterize transitions between those states (Rabiner, 1989; Singh et al., 2011). However, limited research has integrated ECT and S-O-R with latent-state modeling of actual continuous learning behavior in an LMS-centered university setting. This study addresses that gap using ordered observations from M University. Its scope is deliberately limited to the LMS, analytics dashboards, behavioral tracking plug-ins, digital instructional content, and teacher/peer interaction; it does not generalize the findings to unobserved VR, AR, or metaverse environments. Accordingly, the study addresses three research questions:

1. Which technology-enabled instructional conditions and learner appraisals are associated with undergraduates’ continuous learning behavior?
2. What latent continuous-learning states can be identified from repeated behavioral observations, and what transition patterns occur among those states?
3. How do the associations of affective identification, immersion, satisfaction, and attention with continuous learning behavior vary across latent states?

The study makes three contributions. First, it separates actual continuous learning behavior from continuance intention and momentary engagement and specifies affective identification as a distinct organism variable. Second, it integrates ECT’s post-adoption appraisal logic with the S-O-R sequence, mapping observed LMS content and feedback and teacher/peer interaction to internal appraisals and state-contingent behavioral responses. Third, it uses an HMM to characterize latent persistence states and transitions and employs SVR only as an exploratory analysis of state-specific associations. The resulting profiles provide a differentiated basis for instructional support, while the observational design and incomplete archived diagnostics require cautious, non-causal interpretation.

## 2 Constructs, hypotheses development and proposed theoretical model

### 2.1 Affective identification

Affective identification is defined here as a learner’s felt alignment with and acceptance of the technology-mediated teaching process. This specific construct label is used consistently throughout the study. Intelligent tools may strengthen identification when they make feedback, content, and teacher-student interaction feel personally relevant (Ebadi and Amini, 2024; Li et al., 2020; Lin and Chen, 2024). However, this relationship is expected to be contingent on the learner’s current behavioral state because novelty, task demands, and prior experience may change how affective cues are appraised.

*H1*: Affective identification is positively associated with more persistent continuous learning behavior, although the strength of the association may differ across latent states.

### 2.2 Immersion experience

Immersion refers to perceived absorption in the observed LMS-centered and analytics-supported learning environment. Prior research suggests that well-aligned interactive features can focus attention and support persistence, but excessive novelty, interface demands, or cognitive load may interrupt rather than sustain learning. Thus, the expected positive association is theoretically plausible but not assumed to be uniform across learners or states (Shooshtari and Manuel, 2014; Hawes and Arya, 2023).

*H2*: Immersion is positively associated with continuous learning behavior on average, but the direction and magnitude of this association may vary across latent states.

### 2.3 Social influence

Social influence is an external S-O-R stimulus arising from teacher practice, peer feedback, and the norms conveyed through technology-mediated interaction. It can shape learners’ appraisals by signaling whether the learning activity is useful, supported, and socially valued. Its association with persistence should therefore depend on whether these external cues translate into satisfaction rather than operating as a direct or universal effect (Wardoyo and Firmansyah, 2020; Lin and Yu, 2025).

Learning satisfaction is the learner’s post-use appraisal of the technology-supported learning experience. In ECT, satisfaction follows the confirmation of expectations and is a proximal correlate of subsequent continuance. Here it is modeled as an organism variable that may connect teacher or peer influence to repeated learning behavior, while recognizing that satisfaction and observable persistence are conceptually distinct (Bhattacherjee, 2001; Ullah et al., 2023).

*H3*: Social influence is positively associated with continuous learning behavior through learner satisfaction.

### 2.4 Teaching content and environment

Technology-enabled teaching content and the learning environment are external stimuli. Their relevance, clarity, pacing, and adaptability may support attention, but the association is expected to depend on alignment with learners’ prior knowledge and task demands. The present study therefore focuses on LMS content, analytics-supported feedback, and classroom interaction rather than extrapolating to unobserved VR, AR, or metaverse settings (Mailizar et al., 2021; Dimitriadou and Lanitis, 2023).

Learning attention is an internal organism state reflected in attentive listening, note-taking, thinking, and commenting. It is expected to be a proximal correlate of repeated learning behavior because these actions indicate continuing allocation of cognitive resources. Cognitive preparation and perceived usefulness may support attention, but the strength of these associations may vary across behavioral states (Tabuenca et al., 2021; Ma et al., 2024).

*H4*: Perceived quality of technology-enabled teaching content is positively associated with learning attention, which in turn is associated with more persistent continuous learning behavior.

*H5*: Cognitive preparation is positively associated with perceived usefulness, which in turn is associated with learning attention and continuous learning behavior.

### 2.5 Model construction

In the present study, intelligent technologies refer specifically to the LMS, learning analytics dashboard, behavioral tracking plug-ins, and related data-driven instructional functions used at M University. The construct does not represent every form of artificial intelligence or immersive technology. This bounded definition aligns the theoretical claims with the technologies that generated the empirical observations.

Figure 1 retains the original conceptual architecture but updates the labels to correspond directly with the revised framework. Expectation confirmation and perceived usefulness inform satisfaction within ECT (Bhattacherjee, 2001). The HMM and SVR are analytical procedures rather than theoretical components, and the arrows represent proposed associations rather than causal effects.

**Figure 1:**
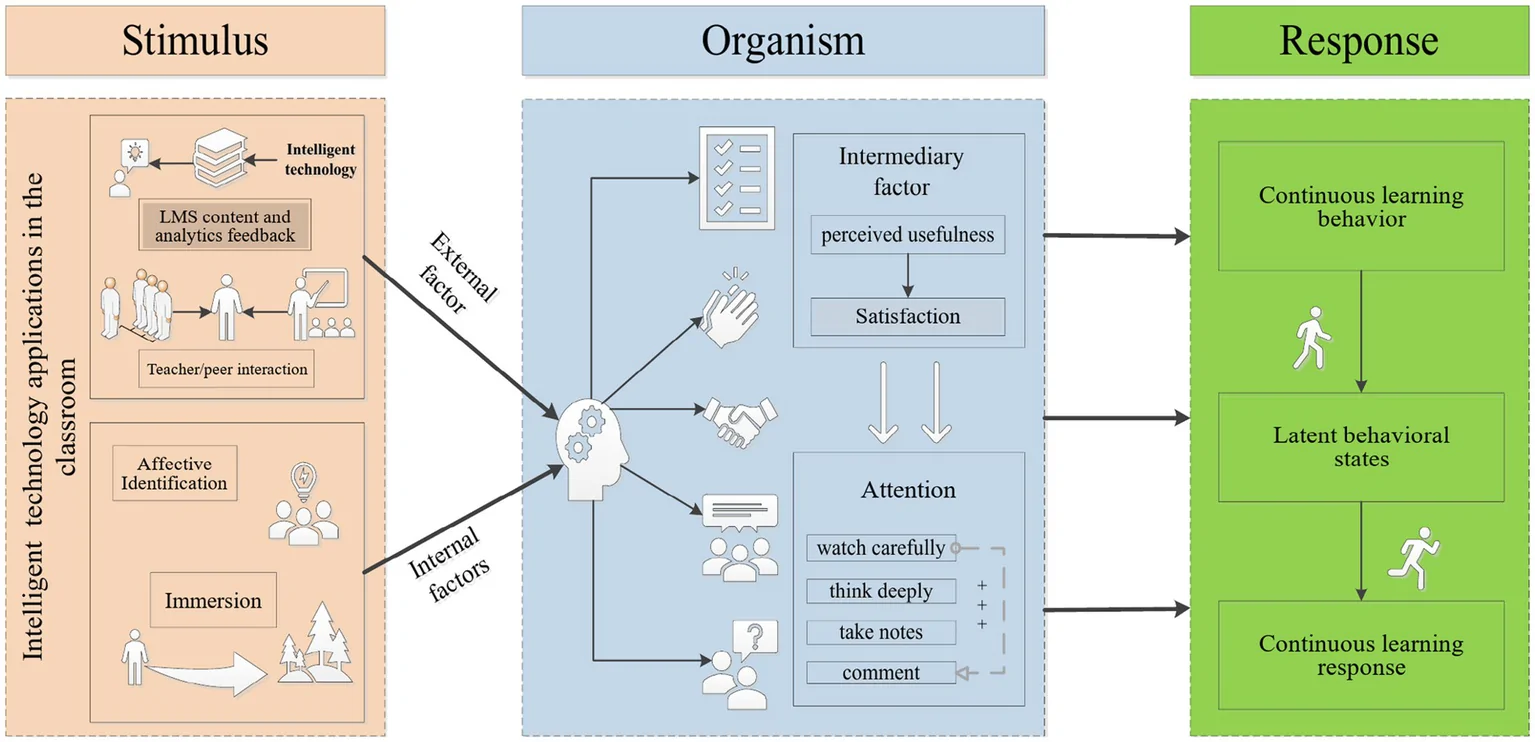
ECT-informed S-O-R conceptual framework. Stimulus (S) comprises LMS content and analytics feedback and teacher/peer interaction; Organism (O) comprises affective identification, immersion, satisfaction, and attention; Response (R) comprises continuous learning behavior and latent behavioral states. The original layout and arrows are retained, with terminology updated. ECT, expectation-confirmation theory; S-O-R, stimulus-organism-response.

## 3 Research methodology

The empirical analysis uses ordered data from an LMS-centered intelligent learning environment. Behavioral tracking plug-ins and server-side scripts recorded assignment activity, commenting, peer interaction, viewpoint endorsement, note-taking, reflection, and attention-related indicators. Psychological variables were derived from the accompanying CCSS questionnaire. The design is observational; consequently, the analysis estimates associations and sequential patterns rather than causal effects of technology exposure.

### 3.1 Hidden Markov models: applicability and methodological comparison

This study examines the determinants of university students’ continuous learning behaviors in intelligent-technology-supported environments, characterizes learning-behavior states and their interconnections, and analyzes how influencing factors operate across states and state transitions. Continuous learning behavior is conceptualized as a temporally evolving process rather than a static outcome measured at a single time point. During this process, learners may occupy qualitatively different phases, yet the underlying learning state is not directly observable and must be inferred from manifest indicators derived from behavioral logs and survey measures. The phenomenon of interest therefore involves unobservable latent states and dynamic transitions among these states over time. An appropriate analytic approach must accommodate measurement error and incomplete indicators while enabling probabilistic inference of latent states and their transitions.

HMM is appropriate because it links repeated observed indicators to unobserved states and estimates switching among those states (Rabiner, 1989; Zucchini and MacDonald, 2009). This is different from latent transition analysis (LTA), which is typically formulated for a smaller number of discrete measurement waves and categorical latent classes. The present data contain ordered within-student observations of unequal sequence length, making an HMM a more direct representation of adjacent-occasion dependence. This advantage does not make HMM universally superior; LTA would be preferable if the research question centered on measurement invariance and class change across a few planned waves. Table 1 clarifies the analytic choice. Regression and conventional SEM can estimate associations among variables but do not make state-transition probabilities their primary estimand. Clustering and latent profile analysis provide cross-sectional groups without a transition model. LTA can estimate class transitions across planned waves, whereas HMM is oriented toward denser ordered sequences and adjacent-state switching. The choice is therefore driven by the data structure and estimand, not by a claim that HMM is categorically superior.

**Table 1:** Methodological comparison for modeling learning states and transition dynamics in continuous learning.

Method	Primary analytic focus	Latent state/class modeling	State-transition probability estimation	Suitability for “learning states–dynamic transitions”
Regression/ML regression	Explanation or prediction of an outcome	No	No	Low
Structural equation modeling	Theory-driven structural mechanisms among latent constructs	Yes	No	Moderate (mechanism testing; not transition modeling)
Clustering/Latent profile analysis	Cross-sectional grouping	Yes	No	Low–Moderate
Longitudinal growth/trajectory models	Continuous trends and trajectory heterogeneity	Yes	Limited (not the core estimand)	Moderate
Latent transition analysis	Class transitions across waves	Yes (classes)	Yes	Moderate–High
HMM	Sequential latent states and switching dynamics	Yes (states)	Yes	High

In sum, HMM is used to uncover the latent-state structure of continuous learning behavior and its transition patterns, while the analysis of influencing factors explains mechanisms associated with state membership and transition tendencies. Together, these analyses form an integrated explanatory framework linking factors, states, and transitions.

### 3.2 HMM modeling approach

An HMM represents each learner’s ordered observations as emissions from an unobserved state process (Zucchini and MacDonald, 2009). The archived script uses hmmlearn GaussianHMM with multivariate Gaussian emissions, diagonal covariance, expectation–maximization (Baum–Welch) estimation, a maximum of 100,000 iterations, and a convergence tolerance of 1 × 10^−5^. Initialization follows hmmlearn defaults; no fixed random_state or multiple-random-start loop is specified in the archived code.

The model comprises an initial-state distribution, a state-transition matrix, and an emission model linking each latent state to the observed behavioral indicators.

λ=(A,B,π)

Where the state transfer matrix A denotes the probability of transferring from one state to another, the observation probability matrix B denotes the probability of observing a certain observation given a certain state, and the initial state probability vector π denotes the probability of the system being in each possible state at t=0 .

1) Initial Continuous Use Behavioral State Probability Vector A .

$A=\{a_{1},a_{2},\cdots a_{N}\}$ , *N* is the number of hidden states, $a_{1}$ is its initial state, and $a_{N}$ represents the initial probability of continuous learning behavior in the Nth state.

2) Continuous learning behavior state transfer probability matrix B .

$$\begin{array}{l} B=B(w,t,t+1)={\left(B_{\mathrm{ij}}^{w}\right)}_{N\times N}\\ B_{\mathrm{ij}}^{W}=p(g_{t}^{w}=j|g_{t}^{w}=i) \end{array}$$ (1)

$B_{\mathrm{ij}}^{W}$ indicates the probability that the student w transfers to the state j at the moment t+1 conditional on moment t being in state i . This formula is used to describe the probability of transfer between student learning states in the teaching process, and the change of each state in the continuous learning behavior is specifically analyzed through parameter estimation (Equation 1).

At adjacent occasions, a learner may remain in the same latent state or move to another. These states are probabilistic, temporary behavioral patterns; they are not assumed to be fixed developmental stages or proxies for academic year.

3) Continuous use behavior decision probability matrix π .

π(w,t) denotes the observable continuous use behavior decision probability matrix of students w at the moment t during the teaching and learning process, which is defined as shown in Equation 2.

$$\pi (w)={\left(\pi_{\mathrm{ik}}^{w}\right)}_{N\times H}$$ (2)

Which $\pi_{\mathrm{ik}}^{w}=p(x_{t}^{w}=v_{k}|g_{t}^{w}=i)$ , $\pi_{\mathrm{ik}}^{w}$ is the probability that a student w generates a continuous learning behavioral decision $v_{k}$ at the moment t based on the state i condition.

### 3.3 Learning satisfaction and attention functions

The satisfaction function is an operational composite that summarizes agreement with teachers or classmates, communication and discussion, and task participation within each latent state. It is not a separate regression model and is not interpreted causally. The causal relationship of the three variables, expressed in terms of utility function as shown in Equation 3.

$$\begin{array}{l} C_{t+1i}^{w}=T_{i1}{\text{Accept}}_{t+1}^{w}+T_{i2}{\text{Interaction}}_{t+1}^{w}\\ +T_{i3}{\text{Participate}}_{t+1}^{w}\;\forall i\in \{1,2,\cdots ,N\} \end{array}$$ (3)

The corresponding weights describe the relative contribution of the observed satisfaction indicators within a state.

The learning-attention function summarizes commenting, note-taking, thinking, and attentive listening at each ordered occasion. These indicators operationalize attention-related behavior rather than a complete measure of cognitive engagement and the causality of the four variables are expressed in utility function as shown in Equation 4 as follows:

$$\begin{array}{l} A_{t+1i}^{w}=S_{i1}{\text{Comment}}_{t+1}^{w}+S_{i2}{\text{Notes}}_{t+1}^{w}+S_{i3}{\text{Reflections}}_{t+1}^{w}\\ +S_{i4}{\text{Watch}}_{t+1}^{w}\;\;\;\forall i\in \{1,2,\cdots ,N\} \end{array}$$ (4)

The weights describe relative indicator contributions. Subsequent analyses examine associations between these composites and state-related outcomes; they do not establish mediation or causality.

### 3.4 Exploratory state influence function

The state influence function combines standardized learning attention, affective identification, immersion, and satisfaction measures into a descriptive state-related score, as shown in Equation 5. This function is an analytical input to the exploratory SVR; it is not an additional theoretical model or an HMM covariate model.

$$\begin{array}{l} E_{\mathrm{ik}}^{w}=\beta_{i1}{\text{Attention}}_{t}^{w}+\beta_{i2}{\text{Emotion}}_{t}^{w}+\beta_{i3}{\text{Immerse}}_{t}^{w}\\ +\beta_{i4}{\text{Satisfy}}_{t}^{w}\forall i\in \{1,2,\cdots ,N\}\; \end{array}$$ (5)

The estimated weights indicate relative predictive association with the state-related score and should not be interpreted as causal effects.

### 3.5 Selection of the number of states using BIC

The Bayesian Information Criterion (BIC; Schwarz, 1978) balances model fit and complexity. Models with one to eight latent states were compared, and the specification with the smallest BIC was retained. BIC is used for relative model selection; it does not by itself establish substantive validity of the resulting labels.

BIC=Kln(n)−2ln(L) (6)

In the BIC expression, *k* is the number of free parameters, *n* is the number of observations, and L is the maximized likelihood. The selected state count was subsequently interpreted using the state-specific indicator profiles and persistence scores.

## 4 Empirical analysis: M University

### 4.1 Data sources, structure, and preprocessing

Data were drawn from M University, a participating institution in the CCSS project, during 2021–2023. Stratified sampling covered academic year, discipline, and gender. After cleaning, 2,133 undergraduates contributed 70,280 recorded observations, equivalent to an average of 32.95 observations per participant; sequence lengths varied across participants. Each ordered record contained a de-identified student identifier, an occasion index, and indicators such as attentive listening, note-taking, critical thinking, agreement, participation, discussion, and commenting.

Preprocessing grouped records by student, ordered them by occasion, coded the four-category frequency indicators numerically, and retained complete indicator vectors for modeling; no model-based imputation is claimed. The analysis then (1) described the measures, (2) compared one- to eight-state HMMs using BIC, (3) interpreted the selected state profiles, and (4) used SVR as an exploratory analysis of state-specific associations. The archived output contains BIC values, state summaries, coefficients, and RMSE values, but it does not preserve random-start comparisons, seed-sensitivity results, the optimization trace, kernel tuning, the train-test split, or convergence logs needed for full computational replication. These missing diagnostics are reported as a limitation rather than reconstructed (Figure 2).

**Figure 2:**
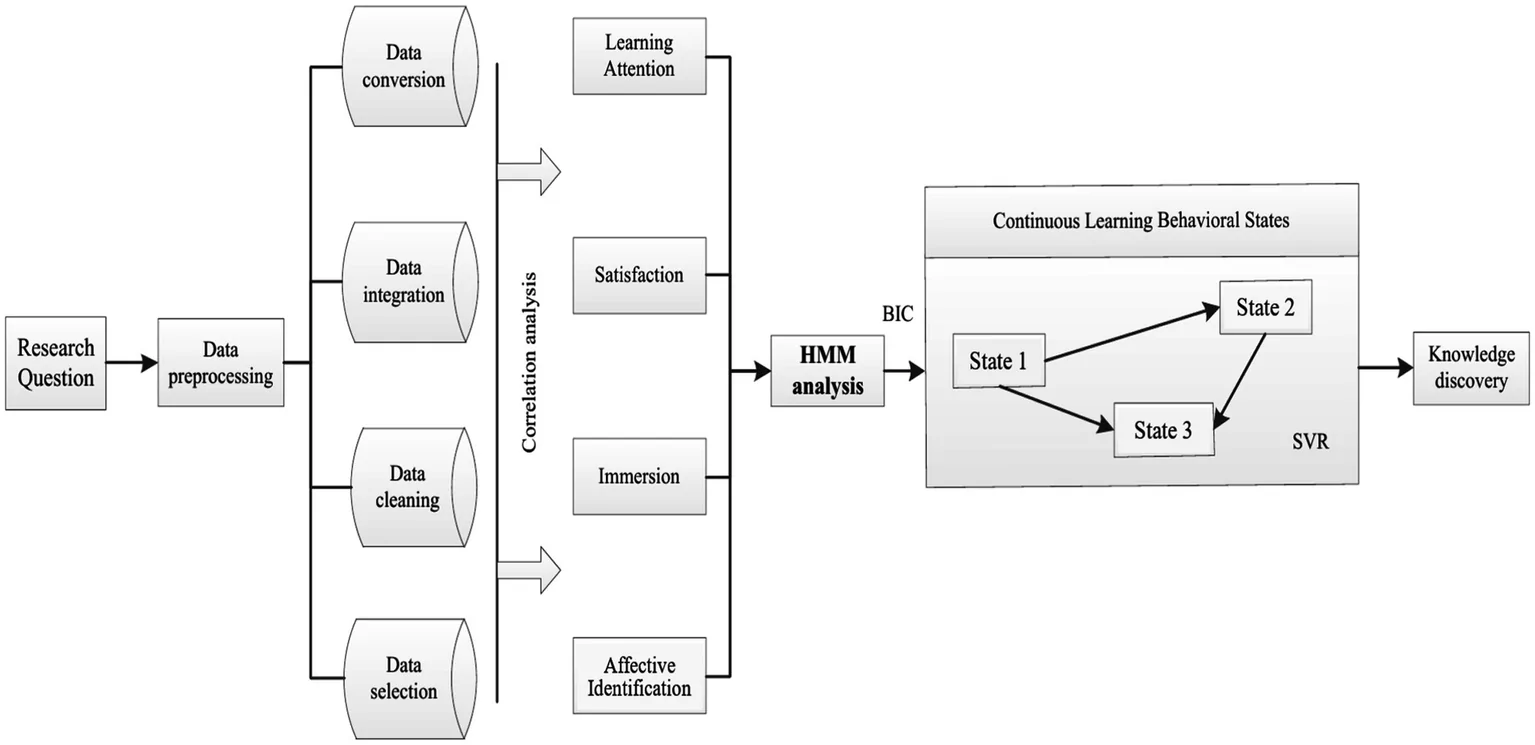
Analysis workflow. The original diagram structure is retained, with harmonized construct and state terminology. HMM, hidden Markov model; BIC, Bayesian information criterion; SVR, support vector regression.

### 4.2 Measurement and variable evaluation

The CCSS questionnaire was administered in Chinese. The English labels below are harmonized reporting translations rather than quotations from a separately validated English instrument; the retained project archive did not contain an independent forward/back-translation log. Items used the four-category response set Always, Often, Sometimes, and Never. Table S1 reports construct roles, sources, and paraphrased sample indicators, while Supplementary Table S6 consolidates item origins, translation information, response format, and measurement indicators.

Table 2 reports the retained item loadings, Cronbach’s alpha, composite reliability (CR), and average variance extracted (AVE). Loadings range from 0.701 to 0.849 and AVE values from 0.642 to 0.774. Cronbach’s alpha ranges from 0.752 to 0.822. However, the reported CR values for immersion (0.642) and learning attention (0.621) are below the conventional 0.70 guideline, so they are described as marginal rather than ‘well above’ threshold. These statistics provide partial convergent evidence, but measurement validity is not regarded as established without complete construct correlations and discriminant-validity evidence.

**Table 2:** Item-level reliability and convergent-validity statistics for the study constructs.

Variables	Items	Loadings	Cronbach’s alpha	Composite reliability (CR)	Average variance extracted (AVE)
Affective identification	EI_1	0.776	0.752	0.768	0.642
	EI_2				
Immersion	I_1	0.701	0.813	0.642	0.774
	I_2				
Satisfaction	S_1	0.823	0.822	0.774	0.668
	S_2				
	S_3				
Learning attention	LA_1	0.849	0.820	0.621	0.648
	LA_2				
	LA_3				
	LA_4				

The archived analysis extract does not contain the construct correlation matrix required to calculate Fornell–Larcker or HTMT discriminant-validity statistics (Henseler et al., 2009). We therefore do not claim that discriminant validity was established and identify this missing analysis as a measurement limitation.

Figure 3 is used only to describe the distribution and spread of ordinal behavioral indicators. Visual frequency patterns do not establish normality, and marginal normality is not a prerequisite for the latent-state process itself. Because the continuous-emission HMM assumes a conditional emission distribution within states, future replication should evaluate alternative emission specifications for ordinal indicators. We removed the prior claim that the variables were normally distributed on the basis of the violin plots.

**Figure 3:**
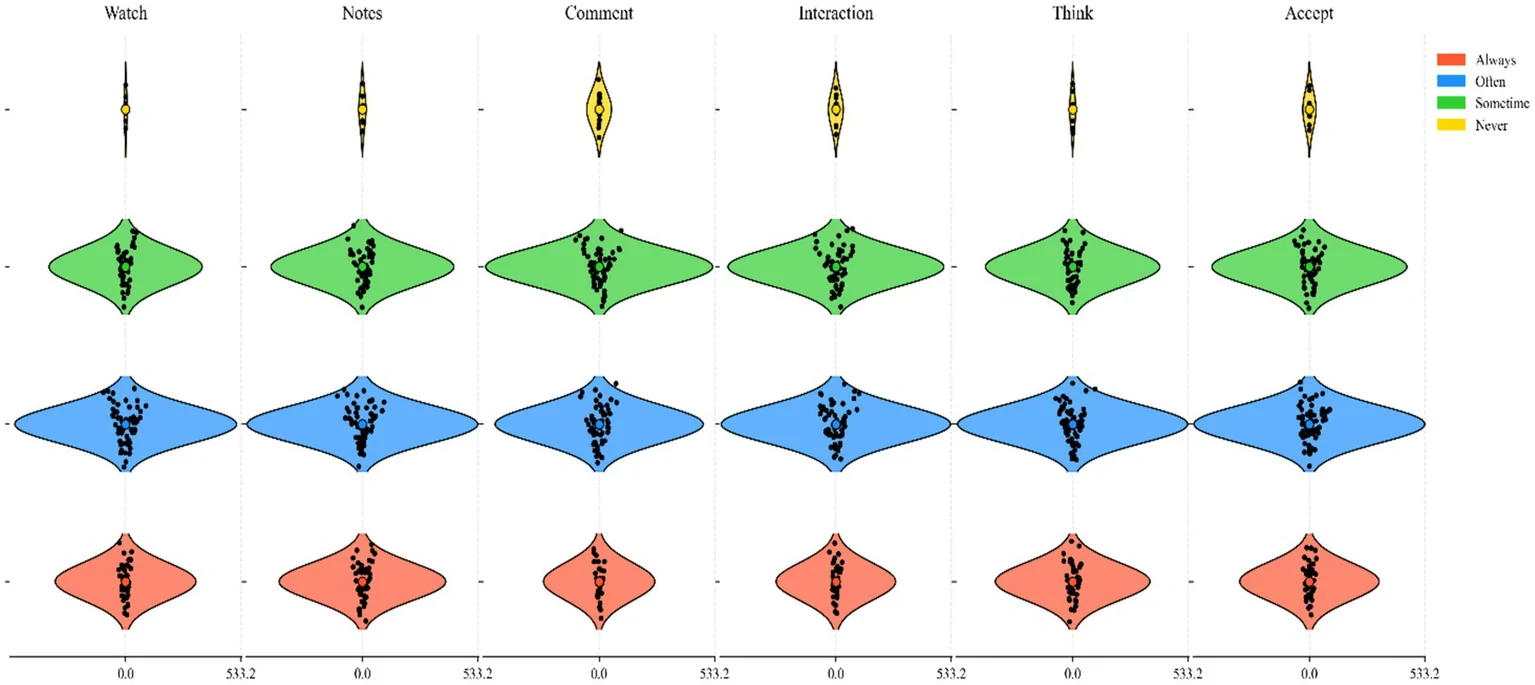
Original embedded distributions of the four-category behavioral indicators (Always, Often, Sometimes, Never), retained unchanged because the plotting source and underlying figure-generation files were unavailable. The figure is descriptive and is not presented as a formal normality test.

### 4.3 HMM training, state selection, and interpretation

Each student’s ordered behavioral records formed an observation sequence. The continuous HMM linked the multivariate indicator vector at each occasion to a latent state. Records with incomplete indicator vectors were excluded during preprocessing, and sequence lengths were allowed to vary. The latent states capture temporary configurations of persistence-related behavior; they do not represent academic year or immutable developmental levels.

Models with one to eight states were fitted with the Baum–Welch algorithm in hmmlearn and compared using BIC (Equation 6). The three-state solution had the smallest BIC (3,326.386). The archived script used default randomized initialization and one fit for each candidate state count; no fixed seed, multiple-start selection, or seed-sensitivity analysis was implemented. Because the fitted model object and optimization trace were not retained, the reported solution remains exploratory and requires replication with fully archived diagnostics.

The states were labeled from their relative indicator profiles and persistence-related scores: State 1, Emerging Persistence; State 3, Established Persistence; and State 2, High Persistence. The nonsequential numbering is retained to preserve the original model output. These labels describe temporary behavioral patterns, not first-, second-, or third-year students (Table 3).

**Table 3:** BIC values for one- to eight-state HMM specifications.

Number of latent states	BIC value	Number of latent states	BIC value
1	4,435.76991775032	5	3,330.31051438144
2	3,365.00844098787	6	3,329.14327503946
3	3,326.38609560417	7	3,413.31051438144
4	3,331.11777805509	8	3,402.68840345487

The retained output is summarized in Table 4. Because the archived initial-state entries and transition rows are not normalized to sum to one, they are reported as comparative scores rather than labeled as probabilities. Valid normalized state and transition probabilities cannot be reconstructed by rescaling the published table entries alone without the fitted model object and decoded sequences.

**Table 4:** Archived HMM state scores and state-specific indicator means.

Variable	Initial-state score	State-transition score			Average value			
		S1	S2	S3	Affective identification	Immersion	Satisfaction	Learning attention
S1	0.120	0.83318	0.00000	0.16682	2.792517	1.60616309	1.535738	1.28571429
S2	0.200	0.00000	0.95392	0.04608	2.9132653	2.12312925	2.858699	2.20572717
S3	0.680	0.03964	0.14640	0.81396	2.0975214	1.99605974	2.177724	1.78979981

State 1 (Emerging Persistence) combines comparatively low satisfaction and attention with higher affective identification. State 3 (Established Persistence) contains the comparatively largest archived initial-state score and a stable mid-range behavioral profile. State 2 (High Persistence) shows the highest satisfaction and attention means. These labels are based on comparative profiles, not on academic-year membership or a deterministic developmental sequence. Decoded state-membership counts and dwell-time sequences were not retained, so student proportions and state-duration characteristics are not reported.

### 4.4 Exploratory association results

#### 4.4.1 State-specific SVR analysis

SVR was used downstream of the HMM as an exploratory nonlinear prediction analysis of the standardized state-related score defined in Equation (5). It was not embedded as a covariate model within the HMM and did not test causal paths or interactions. Table 5 reports normalized coefficients, state-specific coefficients, and RMSE. The archived output does not preserve the kernel, hyperparameters, validation folds, or train-test split; consequently, the SVR results are interpreted descriptively and this reproducibility limitation is explicit.

**Table 5:** Exploratory SVR coefficients and prediction error by latent state.

Variable	Weighting parameter	Normalized coefficient (descriptive)	RMSE	State-specific SVR coefficient		
				State 1	State 2	State 3
Attention	$\beta_{i1}$	0.750616069	0.08283259	0.32529148	0.8008164	0.45859251
Affective identification	$\beta_{i2}$	−0.123088747	0.09185767	−0.8174392	0.02685341	−0.55900709
Immersion	$\beta_{i3}$	−0.187468478	0.08193911	0.19912566	0.09654411	−0.47303561
Satisfaction	$\beta_{i4}$	0.433645972	0.07986028	0.60779776	0.79290873	0.64062379

Learning attention has the largest positive normalized coefficient (0.751), followed by satisfaction (0.434). Affective identification (−0.123) and immersion (−0.187) are negative in the overall normalized summary, with substantial variation across states. RMSE ranges from 0.080 to 0.092. These standardized coefficients are effect-size-like indicators of relative predictive association, not inferential effects; no *p* values or confidence intervals are available from the retained SVR output.

The comparative state profiles are summarized as follows:

1) State 1: Emerging persistence.

This state combines relatively low satisfaction and attention with comparatively strong affective identification. Learners display participation but not a consistently stable pattern. However, the State 1 SVR coefficient for affective identification is negative (−0.817), so this profile does not provide positive support for H1.

2) State 3: Established persistence.

This state shows a stable, mid-range behavioral profile and the largest archived initial-state score. Attention is positively associated with the state-related outcome, whereas affective identification and immersion are negative in the state-specific SVR summary. One possible interpretation is that the value of immersive features depends on their alignment with task demands and learners’ cognitive availability (Kuhail et al., 2022; Hawes and Arya, 2023); this explanation was not directly tested.

3) State 2: High persistence.

This state has the highest satisfaction and attention means and the strongest positive state-specific coefficients for attention (0.801) and satisfaction (0.793). Immersion is close to zero in this state-specific coefficient (0.097), indicating that its relationship is not uniformly positive. The state is interpreted as a temporary high-persistence pattern, not as a senior academic cohort.

The states should therefore be understood as recurring behavioral configurations. The available evidence does not establish a universal or irreversible progression from one state to another.

#### 4.4.2 Interpretation of factors associated with continuous learning behavior

Figure 4 retains the original descriptive trajectories, state segmentation, and plotted values. Only the state titles and variable labels were revised to remove academic-year wording and outdated terminology; no curve or data point was altered.

**Figure 4:**
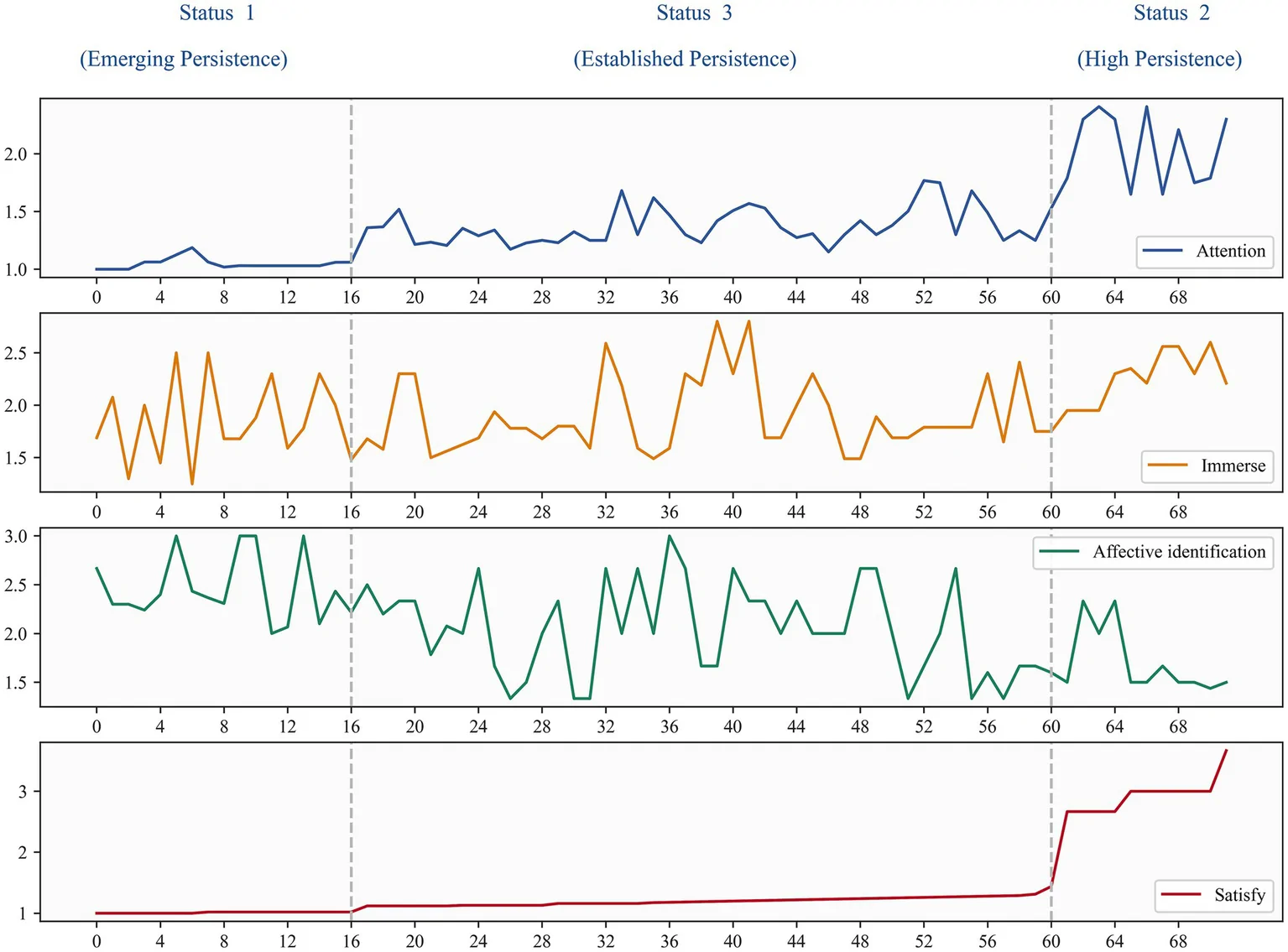
Descriptive trajectories of attention, immersion, affective identification, and satisfaction across Emerging Persistence (State 1), Established Persistence (State 3), and High Persistence (State 2). The original trajectories are retained; only terminology was revised. The display is descriptive and does not establish temporal effects.

The figure and state-specific coefficients support four cautious observations:

First, learning attention and satisfaction show the clearest positive associations with more persistent behavioral patterns. Attention has the largest normalized coefficient (0.751), and satisfaction has the second largest (0.434). These findings are consistent with H4 and H5 at the level of association, but the observational design does not establish that technology-enabled content caused the changes.

Second, the positive association of satisfaction is consistent with the ECT proposition that favorable post-use appraisal accompanies continuance. Social influence may contribute by shaping satisfaction, as proposed in H3, but no formal mediation model was estimated. H3 is therefore regarded as theoretically consistent with the observed pattern rather than causally demonstrated.

Third, the difficulty of moving between state profiles should not be interpreted as proof that external instructional variables produce an upward transition. Instead, the pattern suggests that content quality, teacher guidance, and peer interaction are plausible contextual correlates of attention and satisfaction that should be tested in future covariate-HMM or experimental designs.

Fourth, H1 is not supported overall. Affective identification is negative in the overall summary (−0.123), State 1 (−0.817), and State 3 (−0.559). Only State 2 shows a small positive coefficient (0.027), providing limited state-dependent evidence rather than full support for H1.

Immersion does not support H2 as a uniform positive relationship. Its overall normalized coefficient is negative (−0.187), and the State 3 coefficient is −0.473. We treat this directional reversal as an exploratory finding that may reflect novelty effects, cognitive overload, or mismatch between interface demands and experienced learners’ goals. These explanations were not directly tested and should be evaluated in future research.

## 5 Discussion and conclusion

The findings indicate that continuous learning behavior in an LMS-centered intelligent environment is heterogeneous and dynamic. Attention and satisfaction are the strongest positive correlates of more persistent patterns, while affective identification and immersion vary by state. The results are interpreted as associations among technology-enabled conditions, internal appraisals, and repeated behavior, not as causal effects of intelligent technology.

### 5.1 Research question discussion

#### 5.1.1 Which technology-enabled instructional and psychological factors are associated with undergraduates’ continuous learning behavior?

Intelligent technologies influence students’ continuous learning behaviors through complex interactions between intrinsic and extrinsic factors. While the intrinsic dimension underscores the significance of affective engagement, immersion, cognitive readiness, and participation. Emotional identity facilitates deep connections between students and the learning process. Immersion, enabled by technologies like VR/AR, is suggested to enhance engagement by aligning students’ expectations with actual experiences (Bekteshi, 2025). These emotional and cognitive factors may contribute to deeper learning immersion and sustain long-term learning behaviors. Among external factors, it is the teacher’s proficiency in utilizing intelligent technologies and social influences such as peer feedback that appear to play a pivotal role in shaping students’ learning intentions. Meanwhile, the combined influence of teacher impact and optimized learning environments powered by intelligent tools could be associated with shaping students’ perceptions of technological efficacy, thereby potentially contributing to their learning satisfaction and sustained engagement (Sabri et al., 2024). Furthermore, through the diversity of content and personalized services delivered by intelligent technologies, student participation and learning motivation might be significantly enhanced. This convergence of intrinsic and extrinsic factors likely influences students’ immediate learning experiences and may provide a foundation for continuous, proactive engagement throughout their educational journey.

#### 5.1.2 Which latent continuous-learning states can be identified from repeated behavioral observations, and how do learners move among these states?

This research identifies three distinct State of continuous learning behavior among students within intelligent technology environments, each characterized by unique features and interrelated dynamics. State 1 represents undergraduate students, who are primarily a junior student cohort. It is characterized by significantly lower satisfaction and emotional identity, which leads to behaviors lacking stability and consistency. The probability of transitioning from State 1 to either State 2 or 3 is relatively high, which indicates that students in this State are more likely to shift toward high-engagement learning behaviors (Table 4). State 3, representing a middle-level State, encompasses the largest cohort of students characterized by stabilized and connected learning behaviors. This group exhibits minimal susceptibility to fluctuations in external variables such as immersive experiences or emotional identification, although it responds positively to heightened learning engagement (Caldas et al., 2020). by comparison, State 2 is characterized by strong intrinsic learning motivation, in which students are primarily influenced by satisfaction and learning focus. The low transition probability of this State indicates that continuous learning behaviors are likely to exhibit high stability and persistence.

The interrelationships between these continuous learning behavior states further validate the evolutionary path of learning behaviors, specifically the transition from junior to senior State. This process is influenced by various factors, such as emotional identification, learning satisfaction, and attention. Higher levels of satisfaction and focus can promote sustained engagement in learning activities. These findings explain the dynamic evolution of students’ continuous learning behaviors and suggest that intelligent technologies can stimulate varying levels of learning engagement based on students’ emotional and cognitive levels.

#### 5.1.3 How do the associations of affective identification, immersion, satisfaction, and attention differ across latent states?

Intelligent technologies intervene in university students’ continuous learning behaviors through a coupled mechanism of external facilitation and internal regulation. Significant asymmetry exists among influencing factors across different State (Figure 4). Learning attention and satisfaction serve as primary dependent drivers for students’ continuous learning behaviors. These factors facilitate transitions from junior State and play a decisive role in sustaining senior State learning behaviors, thereby helping students develop stable and highly participatory learning habits. However, transitions between continuous learning State are difficult to achieve through internal variables alone. High-quality intelligent technology applied to instructional content design, task structure, and teacher coordination serves as a potent external catalyst (Zhang et al., 2025). It may enhance attention but also converts perceived utility into satisfaction, thereby facilitating upward transitions in continuous learning State. Salam et al. (2025) demonstrated, building upon the revised theoretical model of acceptance of Revised Artificial Intelligence Device Usage (RAIDUA), that positive satisfaction emotions exert a moderating effect on interactional behavior between users and actual AI devices. Interactions among influencing factors exhibit nonlinearity and role reversal: In the junior State, affective resonance appears to exert a significant positive effect. Students in this stage experience rapid engagement through emotional connection with intelligent technologies and instruction. Conversely, in the senior State, immersive experiences are likely to become neutral or even counterproductive. Overloaded intelligent media resources may trigger skepticism among senior State learners, leading to shifts in learning behaviors. Weight coefficients from the SVR analysis elucidate this mechanism. Table 5 reveals that learning attention and satisfaction contribute most significantly to continuous learning behavior with positive effects, particularly in State 2. Conversely, immersive experience and affective identification show opposing effects as State transition, indicating that internal factors may exert regulatory rather than uniform influence. This finding provides further scientific evidence that students can be seen as a complex collection of State during the learning process. Consequently, instruction should implement targeted teaching and guidance tailored to students in different State.

In summary, intelligent technologies do not uniformly enhance student learning outcomes but rather appear to reconfigure the control parameters of technology-supported systems. External quality improvements are likely to boost students’ learning engagement, thereby generating stable learning satisfaction. Internal factors serve as mediators, sometimes even producing inhibitory effects that might make students prone to developing learning path dependencies. Therefore, integrating intelligent technology tools with teacher practices and customizing the design based on the characteristics of different continuous learning behaviors is essential to further enhance the added benefits of technology-supported education.

## 6 Conclusion

Key findings. Using 70,280 recorded observations from 2,133 undergraduates, the analysis identified three latent continuous-learning patterns. Attention and satisfaction were the strongest positive correlates of persistence, whereas affective identification and immersion showed state-specific relationships. H1 was not supported overall and received only limited state-dependent evidence in State 2; H2 was not supported as a universal positive hypothesis because immersion was negatively associated with the Established Persistence profile. Theoretical and methodological implications. The study distinguishes actual repeated behavior from continuance intention and momentary engagement, integrates ECT with S-O-R, and demonstrates the value of latent-state modeling for questions that cross-sectional methods cannot address directly. At the same time, incomplete archived diagnostics and non-normalized state-score reporting mean that the HMM and SVR findings should be treated as exploratory until replicated with fully reproducible model objects and validation procedures. Practical implications. Instructors can use declining participation, infrequent note-taking, reduced reflection, and low satisfaction as signals for an Emerging Persistence profile and respond with clearer task structure, timely feedback, and low-friction participation prompts. For Established Persistence, systems should reduce unnecessary novelty and support goal-directed feedback. For High Persistence, instructors can provide autonomy, advanced tasks, and peer-mentoring opportunities while avoiding excessive prompts. These recommendations are design implications, not validated treatment effects.

### 6.1 Limitations and future research

This study has several limitations. First, the observational design cannot establish that technology, teacher behavior, or internal appraisals caused a state transition. Second, out-of-class learning was not captured. Future research should use multi-institutional samples, discipline- and teacher-level covariates, validated multilingual measures, fully archived code and model objects, alternative ordinal-emission specifications, sensitivity analyses, and experimental or quasi-experimental designs.

## Data Availability

The original contributions presented in the study are included in the article/supplementary material, further inquiries can be directed to the corresponding author.
